# Advances in nanomedicine and delivery systems for gastric cancer research

**DOI:** 10.3389/fbioe.2025.1565999

**Published:** 2025-03-21

**Authors:** Sizhe Wang, Jilei Li, Zhenyu Zhang, Shasha Cao, Zihan Zhang, Yifan Bian, Yanchao Xu, Chunzheng Ma

**Affiliations:** ^1^ Henan University of Chinese Medicine(The Second Clinical Medical College of Henan University of Chinese Medicine), Zhengzhou, Henan, China; ^2^ Henan Province Hospital of TCM, Zhengzhou(The Second Affiliated Hospital of Henan University of Chinese Medicine), Zhengzhou, Henan, China

**Keywords:** gastric cancer, nanomedicine, nanomaterials, nano drug delivery systems, diagnosis and treatment

## Abstract

The early diagnosis rate of gastric cancer is low, and most patients are already at an advanced stage by the time they are diagnosed, posing significant challenges for treatment and exhibiting high recurrence rates, which notably diminish patients’ survival time and quality of life. Therefore, there is an urgent need to identify methods that can enhance treatment efficacy. Nanomedicine, distinguished by its small size, high targeting specificity, and strong biological compatibility, is particularly well-suited to address the toxic side effects associated with current diagnostic and therapeutic approaches for gastric cancer. Consequently, the application of nanomedicine and delivery systems in the diagnosis and treatment of gastric cancer has garnered increasing interest from researchers. This review provides an overview of recent advancements in the use of nanomaterials as drugs or drug delivery systems in gastric cancer research, encompassing their applications in diagnosis, chemotherapy, radiotherapy, surgery, and phototherapy, and explores the promising prospects of nanomedicine in the treatment of gastric cancer.

## 1 Introduction

In 2022, there were over 960 thousand new cases of gastric cancer globally, resulting in approximately 660 thousand deaths, both figures ranking fifth worldwide (Siegel et al., 2022). Projections indicate that by 2050, annual cancer incidences will reach 35 million, marking a 77% increase from 2022 levels (Bray et al., 2024). Gastric cancer, characterized by high incidence and mortality rates, places a substantial burden on healthcare systems and the World Health Organization due to its extensive consumption of medical resources. Gastric cancer has a low accuracy in early diagnosis, suboptimal treatment outcomes, high recurrence rates, and a 5-year survival rate that remains below 20% (Liu Q. et al., 2023). Most gastric cancer patients are diagnosed at advanced stages because early symptoms are often absent or nonspecific, leading to delayed detection (Liu N. et al., 2022; Thrift and El-Serag, 2020; Tian et al., 2020). Current primary treatment modalities include chemotherapy, radiotherapy, surgery, immunotherapy, and phototherapy (Li et al., 2023). While these treatments can effectively eliminate gastric cancer cells to varying degrees, they frequently result in drug resistance and multiple adverse effects such as vomiting and hair loss (Zhu T. et al., 2024). Nano-drug delivery systems, as a novel drug delivery approach, offer the advantage of precisely targeting tumor cells while causing relatively small damage to healthy tissues and cells (Moradian and Mohammadzadeh, 2023).

**FIGURE 1:**
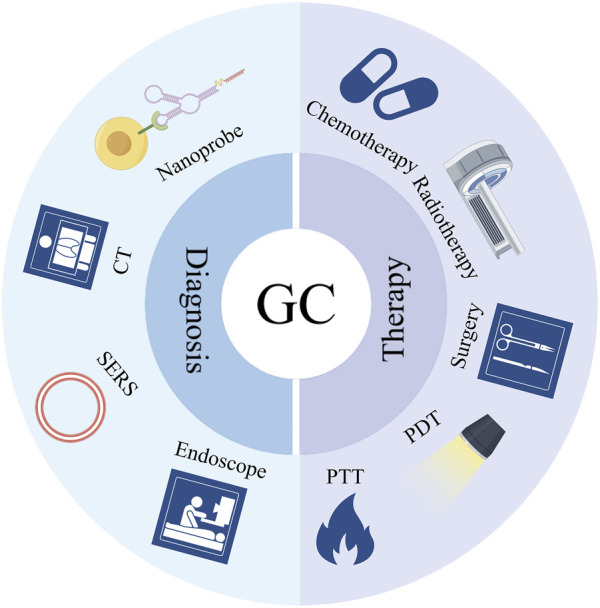
Application of nanomedicine and delivery systems in the diagnosis and treatment of gastric cancer.

Nanomaterials, typically ranging from 1 to 100 nm in size (Jeevanandam et al., 2018), exhibit excellent inclusivity, targeting capabilities, and structural stability (Li et al., 2021; Yang et al., 2022; Yao et al., 2020).In recent years, novel nanotechnology has led to remarkable advancements in nanomedicines, which have made extraordinary achievements in cancer diagnosis and treatment (Bor et al., 2019). Research indicates that nanomaterials hold substantial promise in areas such as early cancer diagnosis, targeted chemotherapy drug delivery, radiation sensitization, surgical tracing, and phototherapy (He et al., 2015). Furthermore, nanomedicine demonstrates strong compatibility with vaccines and 3D printing technologies, opening up extensive prospects for improving the diagnosis and treatment of gastric cancer (Alsharabasy and Pandit, 2024; Ke et al., 2022; Liu Y. et al., 2023).In the diagnosis domain, nanosensors play a pivotal role in detecting cancer at earlier stages, as exemplified by the use of nano-probes (Li et al., 2018). The principle of “early detection and early treatment” has a vital meaning for extending patient survival and enhancing quality of life. (Figure 1).

## 2 Application of nanomaterials diagnosis in the of gastric cancer

Imaging techniques and endoscopic examinations represent a currently prevalent approach for diagnosing gastric cancer. However, these approaches have certain limitations, including blind spots and difficulties in accurate characterization (Li et al., 2021; Yang et al., 2022). Nanotechnology-based diagnostic methods can be divided into direct and indirect approaches. Direct diagnosis involves the use of nanomaterials conjugated with fluorescent substances to form tracers, enabling improved visualization of tumor locations. This provides more precise guidance for subsequent surgical procedures, radiotherapy, and chemotherapy. Indirect diagnosis refers to the integration of nanomaterials into existing diagnostic technologies, such as computed tomography (CT), surface-enhanced Raman scattering (SERS), and endoscopy, to complement existing testing techniques in confirming the diagnosis of gastric cancer.

### 2.1 Direct application of nanomaterials in gastric cancer diagnosis

The application of nanocarriers in the diagnosis of gastric cancer involves the use of nanoscale materials or structures as delivery vehicles to precisely transport diagnostic agents or markers to gastric tissues. This approach facilitates early detection, localization, and assessment of gastric cancer (Kwak et al., 2024). By integrating the benefits of nanotechnology and molecular biology, this technology aims to improve diagnostic sensitivity and specificity, reduce the need for invasive procedures, and enhance patient comfort (Mollasalehi and Shajari, 2021). Key technical requirements include: Size and Permeability: Nanomaterials must be capable of penetrating biological barriers while avoiding clearance by macrophages; Targeting and Loading Capacity: Nanomaterials should be able to conjugate with specific ligands to deliver sufficient diagnostic agents to the target sites; Regulatory Compliance: The design and manufacturing of nanocarriers must comply with relevant pharmaceutical regulations and safety standards, undergoing rigorous safety and efficacy testing (Ruan et al., 2012; Wang et al., 2011). Zhang et al. (2025) devised perovskite-based probes, designated as AZI-PQDs, tailored specifically for the identification of gastric cancer tumors. These probes demonstrated accurate detection of gastric cancer cells in both *in vitro* and *in vivo* fluorescence imaging experiments while ensuring minimal immunogenicity and toxicity. This approach showcases substantial promise for the diagnosis of gastric cancer. Furthermore, Ma et al. (2024) introduced an electrochemical biosensor, termed MoS_2_-Au@Pt, which exhibits remarkable sensitivity and selectivity. Notably, this biosensor can detect miR-19b-3p at exceedingly low concentrations—down to 0.7 a.m.—thus effectively differentiating between normal individuals and those afflicted with gastric cancer. Wu et al. (2021) established a sensitive single-nucleotide polymorphism (SNP) detection system that demonstrated high selectivity and sensitivity for detecting mutations in the p53 gene. This system has the ability to identify early gastric cancer. Zhang C. et al. (2022) developed an electrochemical cell sensor named GO-AuNSs@rBSA-FA for analyzing MGC-803 cells. This sensor quantitatively detects cells in the range of 3 × 10^2^ to 7 × 10^6^ cells/mL, offering the potential for early gastric cancer detection in clinical settings. Shi et al. (2019) introduced a nanoprobe named T-MAN, which targets tumor cells and activates matrix metalloproteinase 2 (MMP-2), enabling the precise *in situ* detection of gastric tumors in a mouse model during surgery for the first time (Wang et al., 2014).

### 2.2 Indirect application of nanomaterials in gastric cancer diagnosis

Nanomaterials can be integrated with existing diagnostic techniques to detect gastric cancer cells. It can be combined with existing CT, SERS, or endoscopy for the diagnosis of gastric cancer. By precisely delivering diagnostic agents via nanocarriers and integrating multiple imaging techniques, this approach enables early diagnosis and comprehensive evaluation of gastric cancer (Cai et al., 2023). Specific criteria cover aspects such as the design of nanocarriers, the performance of imaging techniques, and the ease of operation, ensuring the safety and effectiveness of this method in clinical applications. This not only enhances the accuracy of gastric cancer diagnosis but also provides patients with more treatment options and opportunities.

#### 2.2.1 Application of nanomaterials combined with CT in gastric cancer diagnosis

CT imaging can accurately depict the size and location of gastric cancer cells and is commonly used for preoperative planning and surgical risk assessment. CT offers several advantages, including non-invasiveness (Ma et al., 2022), high image resolution, and the ability to perform three-dimensional reconstruction. However, it has certain limitations, such as reduced sensitivity in detecting small lesions in the early stages (Wei et al., 2024), and radiation exposure from CT scans can pose risks to patients. When combined with CT, nanocarriers should efficiently load CT contrast agents to ensure sufficient contrast during CT scans (Yin et al., 2021). Additionally, CT should have high resolution to visualize the anatomical structures of the stomach and potential lesion areas, facilitating accurate analysis and diagnosis by medical professionals. Shi et al. (2018) investigated a fluorescent copper sulfide nanoparticle (RGD-CuS-Cy5.5), which, when combined with CT, enabled *in vivo* imaging and detection of sentinel lymph node (SLN) metastasis in gastric cancer. Xuan et al. (2019) developed a bismuth nanoparticle cluster probe with enhanced permeability and retention properties. These particles, measuring approximately 25–55 nm, can easily penetrate tumor cells and assist CT in the accurate diagnosis of gastric cancer. Huang et al. (2017) created a nanoparticle composite, Au-BSA-DOX-FA, as a CT contrast agent. This material improves CT imaging clarity and enhances the efficacy of targeted gastric cancer treatment, making it a promising new nanocarrier for drug delivery in gastric cancer diagnosis. Shi et al. (2018) devised a nanoparticle named RGD-CuS-Cy5.5, which targets gastric cancer cells in lymph nodes for non-invasive multimodal imaging, allowing for precise identification of SLN metastasis. Kukreja et al. (2017) developed a porous-shell nanoparticle, AuFe NPs, which exhibits T2 contrast effects and reduces X-ray radiation, making it suitable for CT imaging (Zhang et al., 2013).

#### 2.2.2 Application of nanomaterials combined with SERS in the diagnosis of gastric cancer

SERS has emerged as a promising technique for diagnosing gastric cancer by detecting specific biomarkers in bodily fluids (Zhai et al., 2022). Nanocarriers typically utilize gold or silver nanoparticles as SERS substrates (Xie et al., 2024), which can specifically bind to gastric cancer cells or related molecules, thereby enhancing Raman signals and improving the sensitivity and specificity of the detection (Wei et al., 2016; Xie et al., 2024). However, it remains in the research phase and has not yet been extensively adopted in clinical practice. Consequently, advancing research on SERS-based diagnostic methods for gastric cancer is imperative. For instance, Chen et al. (2016) successfully differentiated between early- and late-stage gastric cancer patients and healthy individuals by analyzing volatile organic compounds in exhaled breath, achieving an accuracy rate exceeding 83%. This approach demonstrates potential as an effective preliminary screening tool. Similarly, Pan et al. (2022) developed a sensor utilizing a nanocomposite material (MoS_2_-AuNSs) to detect exosomes, which carry tumor-specific molecular information, thereby facilitating early cancer diagnosis. Huang et al. (2022) designed an Ag@ZIF-67 sensor that energizes plasmonic nanoparticles, enabling non-invasive, rapid, and user-friendly gastric cancer screening with an accuracy rate of up to 89.83%. Shao et al. (2023) introduced Au/SiNUA, which enhances SERS performance with detection times as short as 18 min, while also exhibiting ultra-sensitivity and specificity, making it advantageous for precise gastric cancer screening. Additionally, Cao et al. (2023) developed a microarray chip based on a gold nanohexagon substrate to construct a SERS spectral recognition model, achieving an accuracy rate exceeding 97.5% in gastric cancer diagnosis, thus highlighting its potential as a clinical diagnostic technology.

#### 2.2.3 Application of nanomaterials combined with endoscopy in the diagnosis of gastric cancer

Endoscopy is a primary diagnostic method for gastric cancer, offering speed and convenience. However, it has notable limitations, including the inability to detect early-stage lesions below its resolution threshold (Han et al., 2020) and risks such as perforation and bleeding. When integrated with endoscopy, nanocarriers should exhibit excellent integration and real-time capabilities. This means that the endoscope must be compatible with nanocarriers and imaging technologies, enabling multimodal imaging while providing real-time imaging data to assist doctors in making rapid diagnoses. To address these challenges, Luo et al. (2023) developed a nanoprobe named M-1, which enables imaging under blue laser endoscopy, simplifying procedures and reducing the risk of errors. In animal experiments, the M-1 probe demonstrated exceptional capability in identifying gastric cancer cells, marking a significant advancement in the visualization of blood vessels for early gastric cancer diagnosis. Harmsen et al. (2019) integrated SERS nanoparticles into endoscopy, successfully detecting gastrointestinal tumor cells in animal models. Furthermore, Du et al. (2018) designed a nanoprobe based on dye-labeled human heavy-chain ferritin, which enhances the accuracy of early gastric cancer cell detection and aids in the endoscopic resection of tumor cells.

## 3 Application of nanomedicines in the treatment of gastric cancer

In clinical practice, the primary treatment modalities for gastric cancer include chemotherapy, radiotherapy, surgery, and phototherapy (Li et al., 2024; Wang G. et al., 2024). However, these approaches are often associated with drug resistance and adverse effects such as hair loss and vomiting. (Rai et al., 2021). By utilizing nanoparticles, liposomes, polymers, nano-micelles, and hydrogels as carriers (Cabral et al., 2018; Che et al., 2020; Salapa et al., 2020), nanomedicines can be delivered precisely to tumor cells, minimizing drug loss and increasing drug concentration within the Tumor Microenvironment (TME) (Kaur et al., 2022; Zhu et al., 2022). When combined with existing therapies, nanomedicines have the potential to enhance clinical efficacy in the treatment of gastric cancer.

### 3.1 Application of nanomedicine in chemotherapy for gastric cancer

Chemotherapy remains one of the primary treatment modalities for gastric cancer. However, conventional chemotherapeutic agents often exhibit suboptimal tumor cell targeting and inflict significant collateral damage to normal cells (Zheng et al., 2020a). The side effects of chemotherapy are not only limited to hair loss and gastrointestinal disturbances but also include cardiovascular and liver-kidney dysfunction. Notably, anthracyclines, a class of chemotherapeutic drugs, are associated with severe cardiotoxicity (Geerts et al., 2024), which can manifest as myocardial ischemia in mild cases and heart failure in severe cases, thereby severely compromising the patient’s quality of life. To address these challenges, researchers have focused on developing various nanomaterials to serve either as chemotherapeutic agents or as drug delivery systems. These nanomaterials enhance the precision of drug delivery through mechanisms such as active targeting and passive targeting while being engineered to account for critical factors like pH, temperature, light, and magnetism (Yu et al., 2022). These factors significantly influence drug efficacy and delivery, paving the way for safer and more precise chemotherapy in the treatment of gastric cancer.

#### 3.1.1 Active targeting

Active targeting represents one of the primary strategies for delivering nanomedicine to tumor cells (Garcia-Pinel et al., 2019). This mechanism involves tethering tumor-specific molecular recognition ligands to the surface of nanomedicine, thereby enabling targeted delivery to tumor cell surfaces. The number, spatial distribution, and other parameters of the targeting ligands on the nanomaterials directly influence the targeting efficiency of the nanomedicine. Typically, tumor cells overexpress a repertoire of surface receptors that can bind to these ligands, leading to endocytosis. Kong et al. (2024) developed a multifunctional pH-responsive nano platform named CPP10-PEG@Cur@FT, which delivers curcumin (Cur) to gastric cancer cells through a synergistic combination of chemotherapy and PDT. This platform significantly enhanced the cytotoxicity of Cur against gastric cancer cells. Zhang et al. (2020) demonstrated that Salidroside potentiates the chemosensitivity of apatinib in gastric cancer, and their engineered nano drug delivery system, iVR1-NPs-Apa/Sal, precisely targets tumor cells. Alam et al. (2022) synthesized curcumin-loaded poly (lactic-co-glycolic acid) nanoparticles (Cur-NPs) using a single-emulsion solvent evaporation method. Experimental results revealed that, compared to cells treated with free Cur, a higher number of apoptotic cells were observed after 72 h of Cur-NPs treatment, highlighting the therapeutic potential of Cur-NPs in gastric cancer.

#### 3.1.2 Passive targeting

**FIGURE 2:**
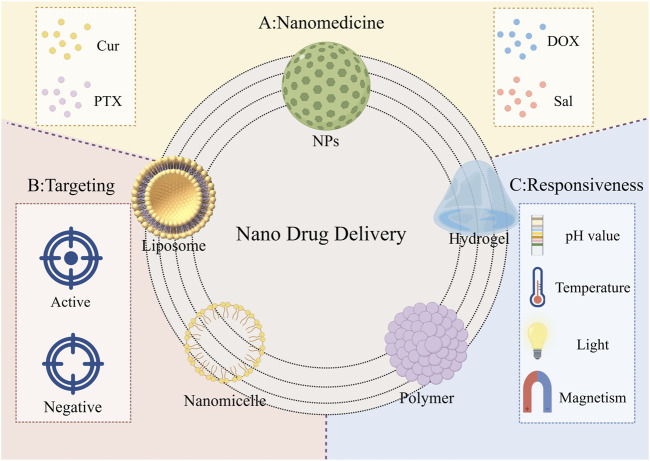
The application of nanomaterials in chemotherapy for gastric cancer.

Passive targeting of nanomedicine to tumor cells is based on the enhanced permeability and retention effect (Obata and Hirohara, 2023). Research has shown that paclitaxel (PTX) synergizes with the AKT inhibitor capivasertib to exert potent antitumor effects, demonstrating significant potential in advanced gastric cancer treatment (Song et al., 2024). Jian et al. (2020) engineered GX1-modified nanoliposomes named GX1-PTX-NLCs, which not only significantly reduced PTX toxicity but also enhanced its antitumor efficacy, making it a promising nano-drug delivery system. Diniz et al. (Diniz et al., 2023) created “Foretinib”-loaded nanoparticles containing tyrosine kinase inhibitors, which exhibited the ability to slow tumor progression and inhibit cell proliferation *in vivo* studies, underscoring their potential as a therapeutic strategy for gastric cancer. Zhang A. et al. (2022) developed dual-loaded DNA micelles (Cur@affi-F/GQs) by incorporating Cur and 5-fluorodeoxyuridine into affi-F/GQs micelles. *In vitro* studies revealed that Cur@affi-F/GQs significantly enhanced the activity of apoptosis-related proteins in the Bcl-2/Bax-caspase eight and 9-caspase three pathways, exerting robust cytotoxic effects on N87 cells. Abedi et al. (2023) synthesized poly (ethylene glycol) -coated nanoparticles that retained 39% of the drug payload after 72 h, demonstrating superior drug retention and advancing the application of nanomedicine in chemotherapy. Zheng et al. (2020b) developed PLGA-NPs co-delivering doxorubicin (DOX) and nitrofurazone, which inhibited gastric cancer cell proliferation by downregulating STAT3 pathway phosphorylation. Chen et al. (2023) engineered a nano-micelle based on carboxymethyl alginate, which, in both *in vitro* and *in vivo* studies, activated cytotoxic T lymphocyte (CTL) activity and polarized M2 phenotype to M1 phenotype, phenotype thereby exerting synergistic antitumor effects and extending the overall survival of gastric cancer animal models. Cur, a traditional Chinese medicine monomer, not only acts as an effective radiosensitizer but also holds promise as a therapeutic agent for gastric cancer. Yaghoubi et al. (2021) targeted human gastric adenocarcinoma cell lines using a combination of Cur and DOX loaded onto carboxylated graphene oxide (APT-CGO), which demonstrated significantly higher inhibitory effects compared to CGO-based drugs (Figure 2).

### 3.2 Application of nanomedicines in radiotherapy for gastric cancer

Radiotherapy is a crucial method for treating malignant tumors, effectively eradicating tumor cells. However, it can also damage normal cells, leading to side effects such as hair loss, gastrointestinal reactions, and bone marrow suppression. Given these side effects, the development of more benign and precisely localized nanomedicines for radiotherapy is crucial. Current nanotherapeutic agents, characterized by reduced toxicity, can be primarily categorized into two major types: organic and inorganic nanoparticles (Wei et al., 2022).

#### 3.2.1 Application of organic nanomedicines in radiotherapy for gastric cancer

Organic nanoparticles are typically used as radio-sensitizers to enhance the sensitivity of tumor cells to radiation. Numerous studies have confirmed that Cur is an effective organic nanoparticle, significantly improving radiation efficiency. Cur exhibits several beneficial properties, including inhibiting tumor cell proliferation, inducing apoptosis in gastric cancer cells, regulating the cell cycle to arrest cells in the radiation-sensitive G2/M phase, and displaying antioxidant effects by scavenging oxidative free radicals generated during radiotherapy. These effects reduce damage to normal cells (Liu Z. et al., 2022). Nosrati et al. (2022) developed a metal-semiconductor heterojunction nanoparticle (Bi_2_S_3_@BSA-Au-BSA-MTX-Cur) loaded with Cur and methotrexate (MTX). This nanoparticle, when administered in a single dose of combined chemoradiotherapy with X-rays, completely eradicated tumors in animal models within approximately 20 days, demonstrating an extremely effective anti-tumor approach. Delahunty et al. (2022) demonstrated that 7-dehydrocholesterol can act as a sensitizer to induce reactive oxygen species (ROS) responses. Encapsulating 7-dehydrocholesterol into nanoparticles (7-DHC@PLGANP) significantly enhances the efficacy of radiotherapy.

#### 3.2.2 Application of inorganic nanomedicines in radiotherapy for gastric cancer

A hypoxic microenvironment (HME) can reduce the sensitivity of tumor cells to radiotherapy. Inorganic nanomaterials are generally used directly in radiotherapy, converting light energy into heat to enhance local radiation or improving the HME of tumor tissues by generating ROS to enhance radiotherapy effects. Unlike traditional radiotherapy, inorganic nanomaterials exhibit excellent biocompatibility and safety, can stably exist within the human body without easy degradation, and have significantly lower toxicity compared to existing radiotherapy methods. Tang et al. (2021) developed an oxygen-generating nanomotor (pHPFON-NO/O_2_) based on hybrid semiconductor organic silicon, which can alleviate HME to enhance the efficacy of radiotherapy. Broekgaarden et al. (2020) demonstrated that the surface chemical design of ultra-small gold nanoclusters (AuNC) enabled high-sensitivity near-infrared and short-wave infrared imaging. They found that combining high-content imaging assays significantly enhances the effectiveness of radiotherapy. Chen et al. (2021) combined Au-OMV with radiotherapy, which not only increased radio-sensitivity but also modulated immune function. In animal experiments, mice treated with Au-OMV in combination with radiotherapy had longer survival times. Wang Z. et al. (2024) designed a tungsten-based nano radiosensitizer (PWAI) to address immune suppression caused by severe MYC upregulation due to radiotherapy. PWAI significantly enhanced the anti-tumor immune response to radiotherapy, reducing the cytotoxicity of T lymphocytes in animal models. Wang H. et al. (2023) developed a nanosystem called MON@pG, which enhanced the radio-sensitivity of gastric cancer cells through ROS-mediated effects, induced mitochondrial dysfunction, and ferroptosis, thereby improving the effectiveness of radiotherapy for gastric cancer.

### 3.3 Application of nanomedicines in surgical treatment of gastric cancer

Surgery remains a crucial treatment for gastric cancer. However, it also has drawbacks such as significant bodily trauma and a long postoperative recovery period (Skapars et al., 2023). Nanomedicine and delivery systems can precisely target tumor cells, reduce incisions, and shorten recovery times. Peritoneal metastatic nodules in gastric cancer, characterized by focal diffusion, small size, and close contact with adjacent organs, are often challenging to identify and completely remove during surgery, frequently leading to surgical failure and cancer recurrence (Guo et al., 2023). Indocyanine green (ICG)-mediated fluorescence imaging is widely used in gastrointestinal surgery. ICG’s excellent permeability and retention properties enable precise and personalized radical surgery (Sun et al., 2024). Wang et al. (2018) synthesized a novel gold nanoshell conjugated with ICG (I-GSN) to overcome the weak targeting ability and poor tissue penetration of chemotherapeutic drugs, as well as incomplete tumor clearance. I-GSN-mediated near-infrared imaging provides sufficient optical contrast for preoperative guidance and intraoperative tumor resection. Wang H. et al. (2023) developed a near-infrared fluorophore methylene blue probe to assist surgeons in clearly visualizing resection margins during gastric cancer surgery. Liposomes, primarily composed of phospholipids, are one of the most commonly used nanocarriers. Encapsulation in liposomes can improve the solubility and stability of anti-cancer drugs (Zhou et al., 2023). Seo et al. (2023) demonstrated that liposome-encapsulated ICG can remain in gastric cancer tissue for a longer period, aiding in lesion marking and SLN identification. Wang et al. (2022) developed a hollow virus-mimetic MnO_2_ nanoshell, which, when used to create the nanoprobe PbS@CdS, can adhere to tumor cells and assist in detailed tumor model investigations under NIR-IIb fluorescence imaging, guiding tumor surgery.

### 3.4 Application of nanomedicine in photodynamic therapy for gastric cancer

Photodynamic therapy (PDT) induces apoptosis in target cells by disrupting the structure and function of organelles through the use of photosensitizers and laser irradiation at specific wavelengths (Tanaka et al., 2021). Due to its high selectivity, minimal invasiveness, and high safety, PDT is widely applied in tumor treatment (Shen et al., 2022). Nanomedicine can enhance the targeted penetration of photosensitizers in PDT, making its integration into PDT a viable option. Current research indicates that PDT has been explored for gastric cancer treatment (Zhang Y. et al., 2023). Yang et al. (2019) demonstrated that the nanoparticle CM/SLN/Ce6 releases its loaded drug in a pH-dependent manner, showing superior anti-gastric cancer effects compared to the free drug in both *in vitro* and *in vivo* studies. (Zhu T. et al. (2024) found that IR780-mediated PDT significantly increases ROS levels, effectively inhibits tumor neutrophil ferroptosis, remodels the immunosuppressive TME, and suppresses gastric cancer cell growth. Xin et al. (2022) revealed that AuS-mediated phototherapy enhances photothermal and vapor effects by increasing ROS, rapidly inducing apoptosis, and improving the clinical efficacy of gastric cancer treatment. Mao et al. (2018) developed 5-fluorouracil-doped silk fibroin nanoparticles, which demonstrated ideal tumor-targeting properties when combined with PDT *in vivo* studies, making it a potential option for cancer treatment. Liao et al. (2021) used a two-photon photosensitizer (Ir-OH) to disrupt mitochondrial redox homeostasis. After irradiation with a 730 nm two-photon laser, they observed enhanced phototoxicity of Ir-OH against human gastric adenocarcinoma due to reduced glutathione levels.

### 3.5 Application of nanomedicine in photothermal therapy for gastric cancer

Photothermal therapy (PTT) and PDT are both forms of phototherapy used in the clinical treatment of gastric cancer (Ouyang et al., 2022; Xie et al., 2020). PTT primarily involves materials with high photothermal conversion efficiency to convert light energy into heat, thereby killing tumor cells (Xiong et al., 2020). PTT is known for its high safety, low toxicity, and short treatment duration, and it can be combined with chemotherapy to enhance efficacy and reduce side effects (Sun et al., 2021; Yan et al., 2020). The integration of nanomedicine into PTT has introduced several advantages, such as improved photothermal conversion efficiency, and light-controlled drug release. Yang et al. (2024) synthesized nanoliposomes encapsulating IR780 and EN4, termed Nano-EN-IR@Lip. These nanoliposomes were shown to mediate PTT, which rapidly kills cancer cells, indicating that this nanoliposome system could potentially function as a novel nano-drug delivery platform for the diagnosis and treatment of gastric cancer. Sang et al. (2021) developed a hydrogel named OSA/AHA/BP/PTX, which exhibited excellent photothermal conversion effects at temperatures close to body temperature in both *in vitro* and *in vivo* experiments. The hydrogel, when combined with chemotherapy, demonstrated a significantly enhanced anti-tumor efficacy. Xia et al. (2023) designed extracellular vesicles modified with CDH17 nanobodies, which, when irradiated with PTT, polarized macrophages from the M2 to the M1 phenotype, exerted synergistic anti-tumor effects and presented a promising treatment method for gastric cancer. Chen et al. (2022) developed a platform named PP@MnNPs, which combines magnetic resonance imaging with chemodynamic therapy/PTT, aligning with the trend of integrated diagnosis and treatment. Both *in vitro* and *in vivo* studies demonstrated that this platform could exert anti-tumor effects and induce ferroptosis. Guo et al. (2020) prepared a novel nanotheranostic agent named HMON@CuS/Gd, which selectively induces mild phototherapy in the TME, inhibiting gastric cancer cell growth while ensuring safety. Wu et al. (2016) synthesized CuInS_2_/ZnS nanocrystals using PEGylated liposomes as linkers, which exhibited lower toxicity than pure liposomes and could be used for PTT and PDT in cancer treatment (Table 1).

**TABLE 1:** Application of nanomedicine and drug delivery systems in gastric cancer radiotherapy, surgery, PDT, and PTT.

	Study	Medicine/Material	Tool/Nanoshape	Name
Chemotherapy	Kong et al. (2024)	Cur	pH-responsive nano platform	CPP10-PEG@Cur@FT
	Zhang et al. (2020)	Sal	NPs	iVR1-NPs-Apa/Sal
	Alam et al. (2022)	Cur	NPs	Cur-NPs
	Jian et al. (2020)	GX1,PTX	Nanoliposomes	GX1-PTX-NLCs
	Diniz et al. (2023)	\	NPs	Foretinib
	Zhang et al. (2022a)	Cur, 5-fluorodeoxyuridine	Micelles	Cur@affi-F/GQs
	Abedi et al. (2023)	PTX	NPs	\
	Zheng et al. (2020a)	DOX, Nitrofurazone	NPs	DNNPs
	Chen et al. (2023)	carboxymethyl alginate	Micelles	\
	Yaghoubi et al. (2021)	Cur, DOX	NPs	APT-CGO
Radiotherapy	Nosrati et al. (2022)	Cur, MTX	NPs	Bi2S3@BSA-Au-BSA-MTX-Cur
	Delahunty et al. (2022)	7-DHC	NPs	7-DHC@PLGANP
	Tang et al. (2021)	Si	Nanoeconomizer	pHPFON-NO/O_2_
	Broekgaarden et al. (2020)	AuNC	Nanoclusters	—
	Chen et al. (2021)	Au-OMV	NPs	—
	Wang et al. (2024b)	W	NPs	PWAI
	Wang et al. (2023a)	ICG/GOx	Nanosystem	MON@pG
Surgery	Wang et al. (2018)	I-GSN	Near infrared imaging	—
	Wang et al. (2023b)	MB	Fluoescent probe	—
	Seo et al. (2023)	LP-ICG	—	—
	Wang et al. (2022)	MnO_2_	Fluoescent probe	PbS@CdS
PDT	Yang et al. (2019)	SLN	NPs	CM/SLN/Ce6
	Zhu et al. (2024b)	Icy7	Liposome	LLI
	Xin et al. (2022)	AuS	NPs	—
	Mao et al. (2018)	5-FU、SF	NPs	—
	Liao et al. (2021)	Ir-OH	—	—
PTT	Yang et al. (2024)	IR780、EN4	Liposome	Nano-EN-IR@Lip
	Sang et al. (2021)	PTX	Hydrogel	OSA/AHA/BP/PTX
	Xia et al. (2023)	CDH17	EV	—
	Chen et al. (2022)	PDA	NPs	Pp@Mn NPs
	Guo et al. (2020)	CuS	Nanotheranostics	HMON@CuS/Gd
	Wu et al. (2016)	ZnS	Liposome, nanocrystal	CuInS2/ZnS

## 4 Potential challenges of nanometer materials

### 4.1 Potential impacts of nanomaterials on healthy tissues and the immune system

Nanomaterials exhibit broad prospects for application in the biomedical field, particularly in the diagnosis and treatment of gastric cancer, due to their unique physicochemical properties. However, the potential toxicity of nanomaterials to healthy tissues is a critical aspect that cannot be overlooked, as it is essential for evaluating their biocompatibility and safety (Deng et al., 2022). When nanomaterials enter a biological system, they can affect healthy tissues through various mechanisms, including physical damage, chemical toxicity, and immune responses (Ling et al., 2023). In clinical practice, a comprehensive evaluation of the biocompatibility and safety of nanomaterials requires consideration of their physicochemical properties, the routes of exposure to the biological system, and the mechanisms of biological responses (Souto et al., 2024). Firstly, nanomaterials can penetrate cell membranes, leading to cell membrane damage, which is the most direct form of physical toxicity (Su et al., 2018). Nanomaterials can also interact with intracellular structures, causing organelle dysfunction. Additionally, variations in the physicochemical properties of nanomaterial surfaces can result in the formation of protein coronas, which may alter the biodistribution, clearance rates, and interactions of nanoparticles with cells, thereby affecting their toxicity. Secondly, chemical toxicity is another significant aspect of the impact of nanomaterials on healthy tissues. Active functional groups or components on the surface of nanomaterials can undergo chemical reactions in the body, producing ROS that induce oxidative stress and cell damage (Lerida-Viso et al., 2023). For example, gold nanoparticles and silver nanoparticles can release metal ions that react with thiol groups within cells, causing cytotoxic effects. Lastly, nanomaterials can also elicit immune responses in the human body, including both immunostimulatory and immunosuppressive effects (Qiu et al., 2017). The size, shape, charge, and surface modifications of nanoparticles can serve as signals for immune cell recognition, leading to the activation or suppression of the immune system, which can result in inflammatory and allergic reactions that impact healthy tissues (Qian et al., 2017). For instance, nanomaterials can activate macrophages and dendritic cells, promoting the release of immune cell factors and triggering localized or systemic inflammation.

### 4.2 Challenges of long-term biocompatibility of nanomaterials with living organisms

**FIGURE 3:**
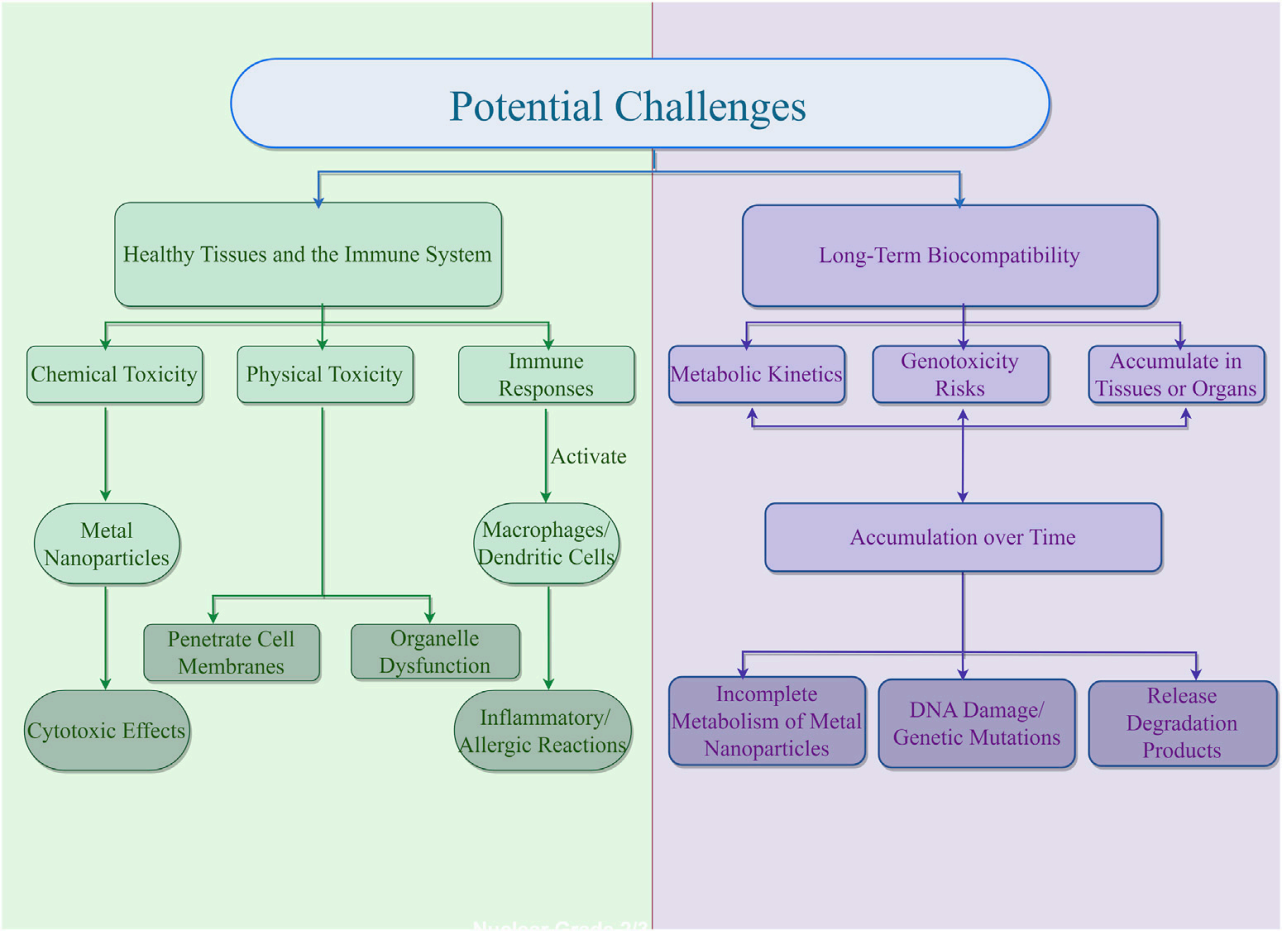
Potential challenges of nanometer materials.

Assessing the long-term biocompatibility of nanomaterials with living organisms is a central challenge, especially in their biomedical applications for gastric cancer treatment. Extended exposure to nanomaterials within biological systems can induce a range of biological effects. Ensuring the safety and efficacy of these materials for gastric cancer treatment necessitates systematic optimization and evaluation to mitigate potential risks associated with prolonged exposure (Parvin et al., 2024). Firstly, biodistribution and metabolic kinetics are critical determinants of the long-term biocompatibility of nanomaterials (Andoh et al., 2024). However, clearance mechanisms can vary depending on material properties, leading to prolonged retention within the body and an increased risk of chronic toxicity (Bizeau and Mertz, 2021). For instance, the degradation and stability of surface modifications influence biocompatibility; unstable modifications may lead to excessive release of degradation products, accumulating toxicity over time. Secondly, prolonged exposure to nanomaterials poses genotoxicity risks (Tang et al., 2024). These materials can directly interact with DNA or induce oxidative stress, leading to DNA damage and genetic mutations. Moreover, sustained contact with certain nanomaterials may alter cellular microenvironments, promoting cancer cell proliferation and metastasis, which negatively impacts gastric cancer therapy. Furthermore, the accumulation of nanomaterials within the body during prolonged exposure is a critical concern. Some nanomaterials may accumulate in specific tissues or organs, increasing their burden (Mahajan and Cho, 2022). For example, metal-based nanomaterials can damage the liver and kidneys through the release of metal ions, whereas polymeric nanomaterials may exhibit long-term toxicity via the release of degradation products (Pan et al., 2023) (Figure 3).

## 5 Promising fields in the development of nano-drugs for gastric cancer

### 5.1 Application of biomimetic nanoparticles in the development of nano-drugs for gastric cancer

Biomimetic nanoparticles are nanoscale carriers constructed by mimicking naturally occurring substances or cellular structures in the biological world. These carriers exhibit excellent biocompatibility and biodegradability, effectively reducing non-targeted effects and enhancing drug delivery efficiency (Anitha et al., 2024). The design inspiration for biomimetic nanoparticles comes from biological structures such as cell membranes, exosomes, and viral particles. By simulating the characteristics of these biological structures, the targeting, biocompatibility, and therapeutic efficacy of nano-drugs can be significantly improved (Xu et al., 2016). Wang et al. (2021) developed a drug delivery tool called the RP (P/T) erythrocyte membrane biomimetic nanosystem. This emerging drug delivery tool, which co-loads PTX and triptolide, can prolong the circulation time of the drug in the blood and evade immune surveillance, making it a promising therapy for gastric cancer. Lei et al. (2023) synthesized a new nano-drug called GIC@HM·NPs with a biomimetic coating, which has the functions of PTT and chemotherapy. This nano-drug not only improves biocompatibility but also allows for more precise drug delivery to tumor sites, enhancing drug utilization efficiency and providing a safer, more efficient, and more precise method for gastric cancer treatment. Zhang L. et al. (2023) designed a co-delivery system called M@BP. In a mouse model of orthotopic gastric cancer, M@BP can effectively target and accumulate in gastric cancer cells, blocking the activation of CDK9 and BRD4 and impeding the growth of gastric cancer cells.

### 5.2 Application of nanotheranostic in the development of gastric cancer nanodrugs

**FIGURE 4:**
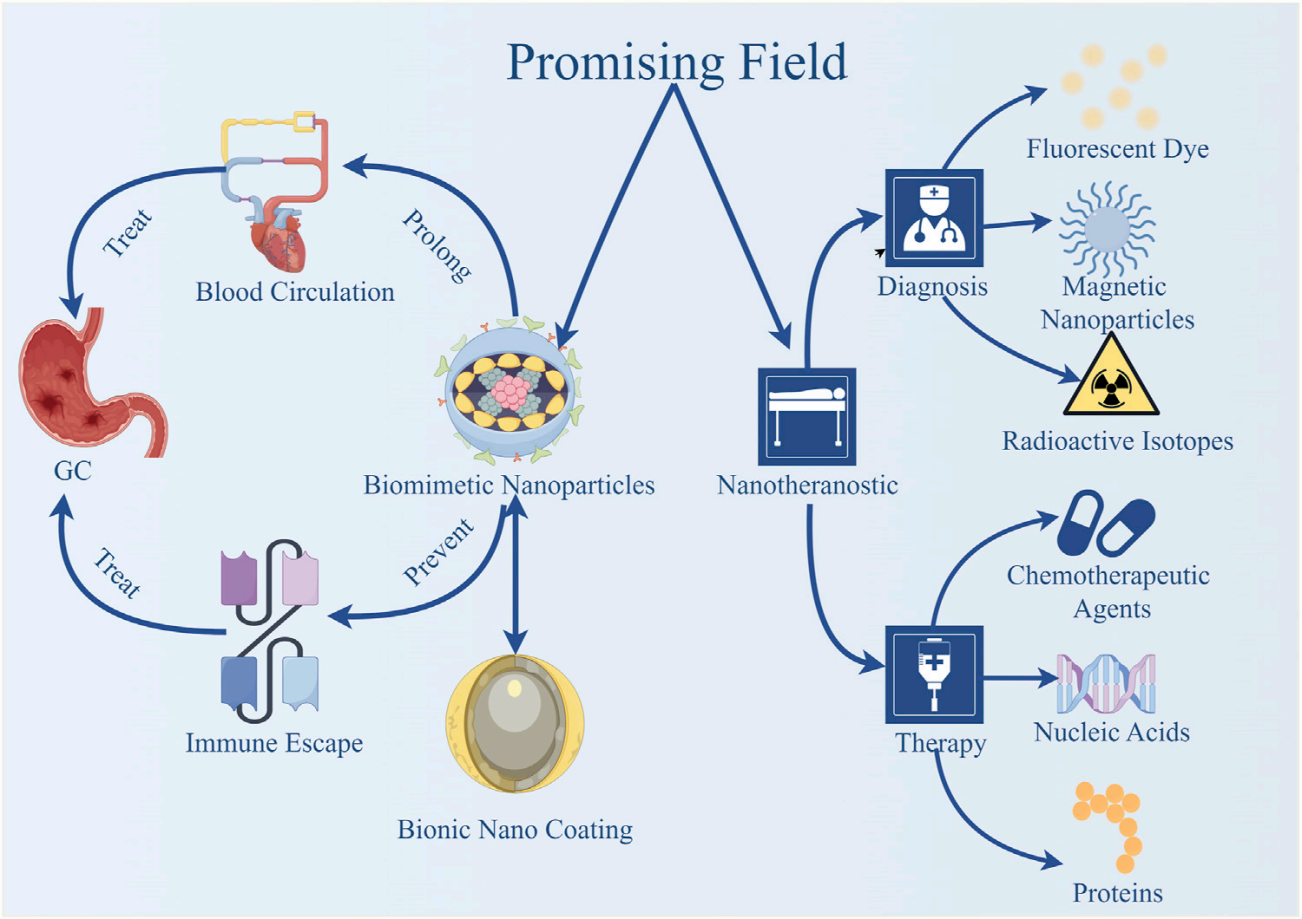
Promising fields in the development of nano-drugs for gastric cancer.

Nanotheranostics is an integrated nanotechnology that combines diagnosis and therapy (Arshad et al., 2021), aiming to construct a multifunctional platform capable of simultaneously achieving disease monitoring and treatment. By co-loading diagnostic molecules, such as fluorescent dyes, magnetic nanoparticles, or radioactive isotopes, and therapeutic molecules, such as chemotherapeutic agents, proteins, or nucleic acids, within biomimetic nanoparticles, it is possible to achieve precise targeting and real-time tracking of gastric cancer, as well as dynamic monitoring of its microenvironment, while simultaneously performing effective targeted therapy (Kumari and Sunil, 2021). Bao et al. (2016) developed a functionalized gold nanoprism named AuNprs, which can be used simultaneously for *in situ* photoacoustic imaging, angiography, and localized thermal therapy. This represents a highly sensitive *in vivo* nanotheranostic platform for detecting gastric cancer tumors and performing targeted therapy. *In vivo* studies have demonstrated that it can reduce tumor size and improve survival rates in mice following localized thermal therapy. Wu et al. (2019) developed a theranostic gold nanoparticle-boron cage assembly, named B-AuNPs, for evaluating the feasibility of boron neutron capture therapy. This study advanced the integrative diagnosis and treatment of gastric cancer by creating detectable boron-containing gold nanoparticles. Zhang et al. (2023a) reported a series of nanobody-derived CD47-targeted agents, among which [^68^Ga]Ga-NOTA-C2 and [^89^Zr]Zr-DFO-ABDC2 exhibited longer circulation times. The study suggests that the optimization of CD47-targeted theranostic approaches may provide new strategies for the treatment of CD47-targeted solid tumors (Figure 4 and Table 2).

**TABLE 2:** Promising areas for nanomedicine development in gastric cancer.

	Study	Medicine/Material	Tool/Nanoshape	Name
Biomimetic Nanoparticles	Wang et al. (2021)	PTX, Triptolide	NPs	RP (P/T)
	Lei et al. (2023)	\	NPs	GIC@HM·NPs
	Zhang et al. (2023b)	\	Black Phosphorus Nanosheets	M@BP
Nanotheranostic	Bao et al. (2016)	\	Gold Nanoprism	AuNprs
	Wu et al. (2019)	Boron	Gold Nanoparticle-Boron Cage	B-AuNPs
	Zhang et al. (2023c)	CD47	Nanobody	[68Ga]Ga-NOTA-C2, [89Zr]Zr-DFO-ABDC2

## 6 Discussion

Gastric cancer, one of the most common types of cancer, continues to have high incidence and mortality rates, placing significant pressure on healthcare systems. While existing treatments have achieved some success, issues such as drug resistance, toxic side effects, and recurrence remain significant challenges. In terms of diagnosis, nanoprobes, due to their large surface area, can easily bind to gastric cancer cells, allowing for the detection of early-stage gastric cancer using fluorescence techniques. The introduction of nanotechnology into gastric cancer diagnosis has the potential to develop more specific and sensitive probes, enhancing diagnostic accuracy. By leveraging the unique physical and chemical properties of nanomaterials, future advancements could integrate optical imaging, magnetic resonance imaging, and ultrasound imaging to achieve multimodal diagnostic fusion, aiding in the accurate staging of tumors. In recent years, significant efforts have been directed toward the integration of diagnosis and treatment in cancer research. A variety of nanomaterials capable of both diagnosing and treating cancer have been developed. These materials enable the initiation of personalized treatment plans based on diagnostic outcomes, thereby minimizing the physical burden on patients associated with multiple diagnostic and therapeutic procedures. In the realm of treatment, nanomedicine has demonstrated considerable promise in several areas, including the targeted delivery of small molecules to tumor cells, the reduction of toxicity, the enhancement of the efficacy of existing treatments, and the improvement of patient quality of life. Nano-drug delivery systems can precisely target tumor cells, increasing drug concentration within the TME, prolonging drug circulation time in the bloodstream, and thereby inhibiting tumor growth, which leads to improved clinical outcomes. Furthermore, nanomedicine can augment the therapeutic effects of PDT and PTT by enhancing the efficiency of light-to-heat and light-to-kinetic energy conversion, aiming to optimize clinical results.

Looking ahead, the development of intelligent nanomedicine and delivery systems represents a key direction for future research. These intelligent systems can monitor changes in the TME in real-time through integrated sensors and adjust drug release accordingly. For instance, temperature- and pH-sensitive nano-drug delivery systems can release drugs under specific TME conditions, thereby achieving targeted therapeutic effects. As nanotechnology continues to evolve, researchers will focus on the development of novel nanocarriers. For example, the use of biocompatible materials such as chitosan as carrier materials can lead to the creation of nanomedicines with higher bioavailability. The integration of novel nanomedicines with advanced drug delivery systems can address diverse treatment needs. The application of nanotechnology in gastric cancer diagnosis and treatment is very promising, but its clinical translation still faces many challenges. For instance, the relationship between the preparation methods, structure, composition, and efficacy of nanomedicines is not yet fully understood; development costs are high, posing significant economic challenges; and many drugs and carriers are still in the research and development phase, not yet meeting clinical standards. Most existing research is still confined to animal experimentation, and the stability of these technologies in the human physiological environment remains uncertain. Researchers should strengthen collaboration with fields such as materials science, chemistry, biology, and engineering, integrating cutting-edge technologies from various disciplines to drive the innovation and development of nanotechnology. For instance, artificial intelligence algorithms can be utilized to optimize the design of nanomedicines, and microfluidic technology can enable high-throughput preparation and screening of nanomedicines. Currently, the most pressing issue to address is the lack of systematic preclinical research and clinical trial data for nanomedicines. Researchers should intensify preclinical studies on nanomedicines, including pharmacokinetic, pharmacodynamic, and toxicological evaluations, to ensure their safety and efficacy in animal models. Additionally, multi-center, large-sample clinical trials are needed to evaluate the efficacy, tolerability, and long-term safety of nanomedicines in gastric cancer patients. Accelerating the clinical translation process of nanomedicines can be achieved through the establishment of standardized clinical trial protocols and data-sharing platforms. Despite the numerous challenges in applying nanotechnology to the clinical diagnosis and treatment of gastric cancer, the continuous advancement of nanotechnology and the deepening of interdisciplinary collaboration are expected to make nanomedicine and delivery systems powerful tools for diagnosing and treating gastric cancer, offering new hope to patients.
