# Supplementary material for: Advances in nanomedicine and delivery systems for gastric cancer research
Source: Front Bioeng Biotechnol. 2025 Mar 21;13:1565999. doi: 10.3389/fbioe.2025.1565999 (PMC11968739; doi:10.3389/fbioe.2025.1565999)
Supplement: Supplementary file 1 [file DataSheet1.docx]

Supplementary Material


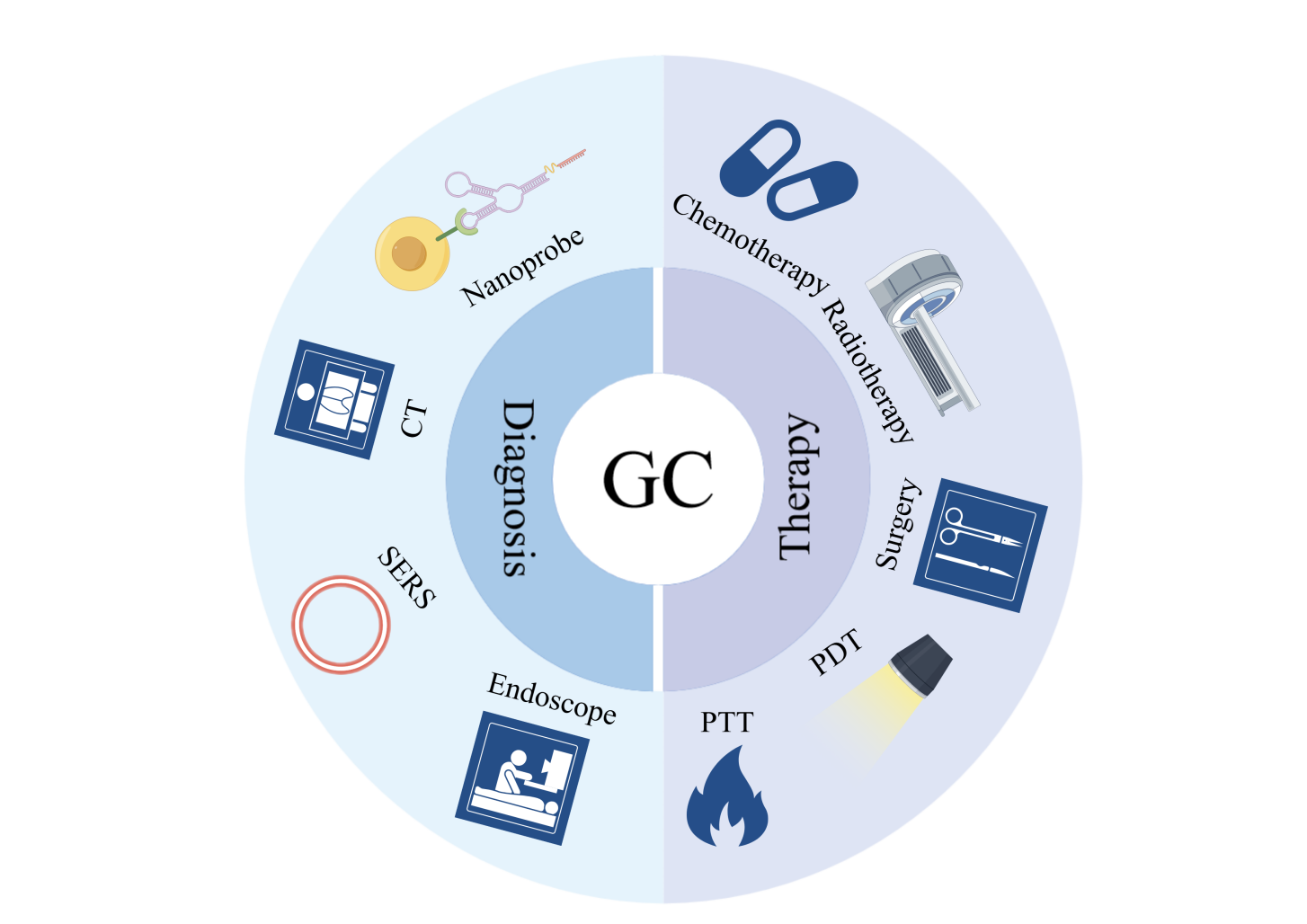


Figure 1. Application of nanomedicine and delivery systems in the diagnosis and treatment of gastric cancer.


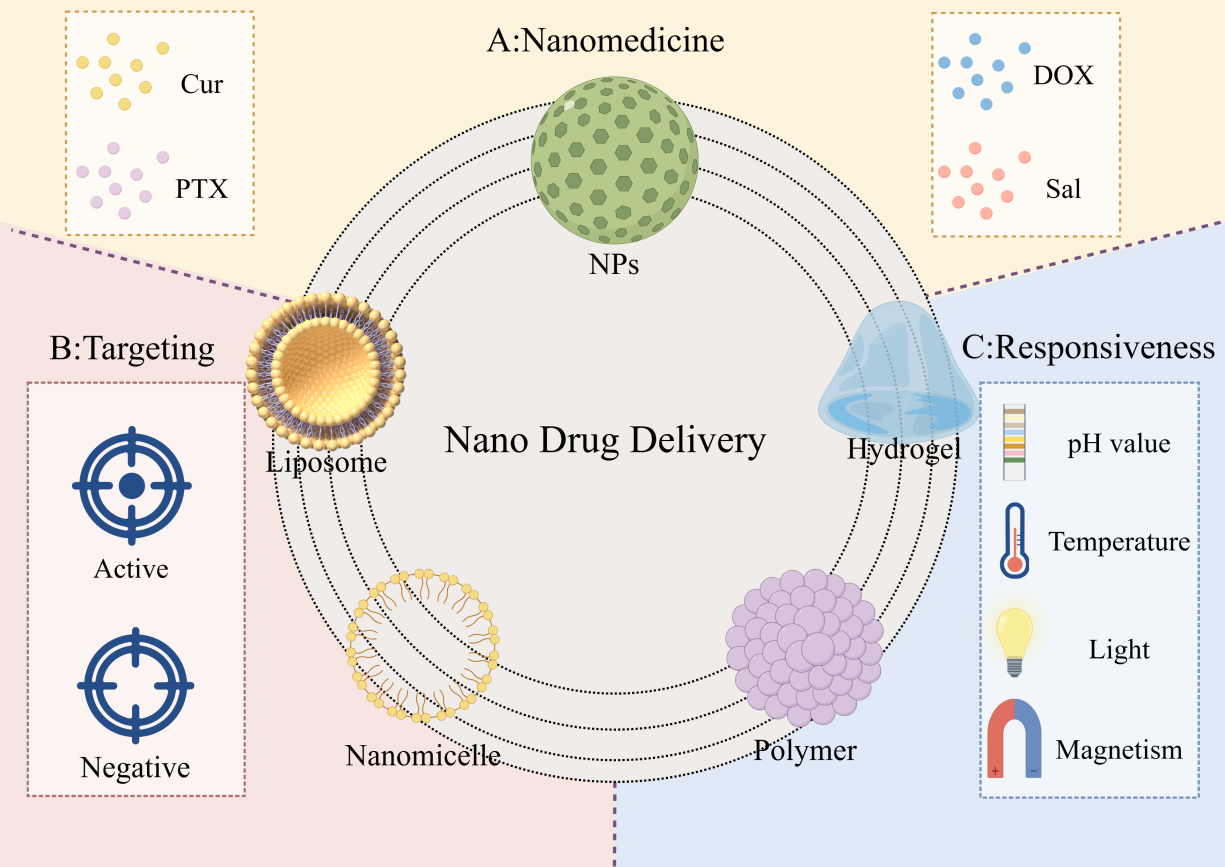


**Figure 2.** The Application of Nanomaterials in Chemotherapy for Gastric Cancer.


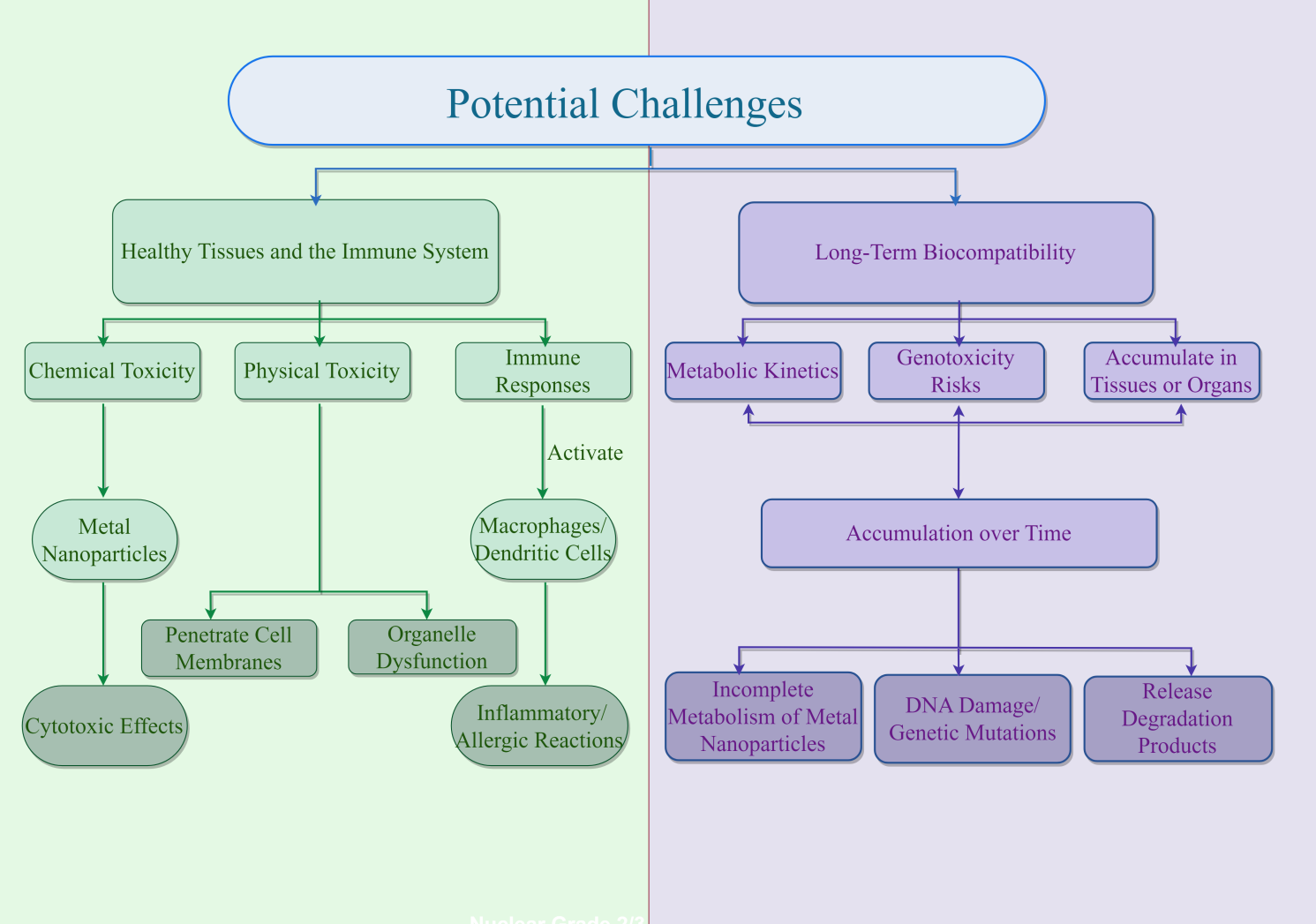


**Figure 3.** Potential Challenges of Nanometer Materials


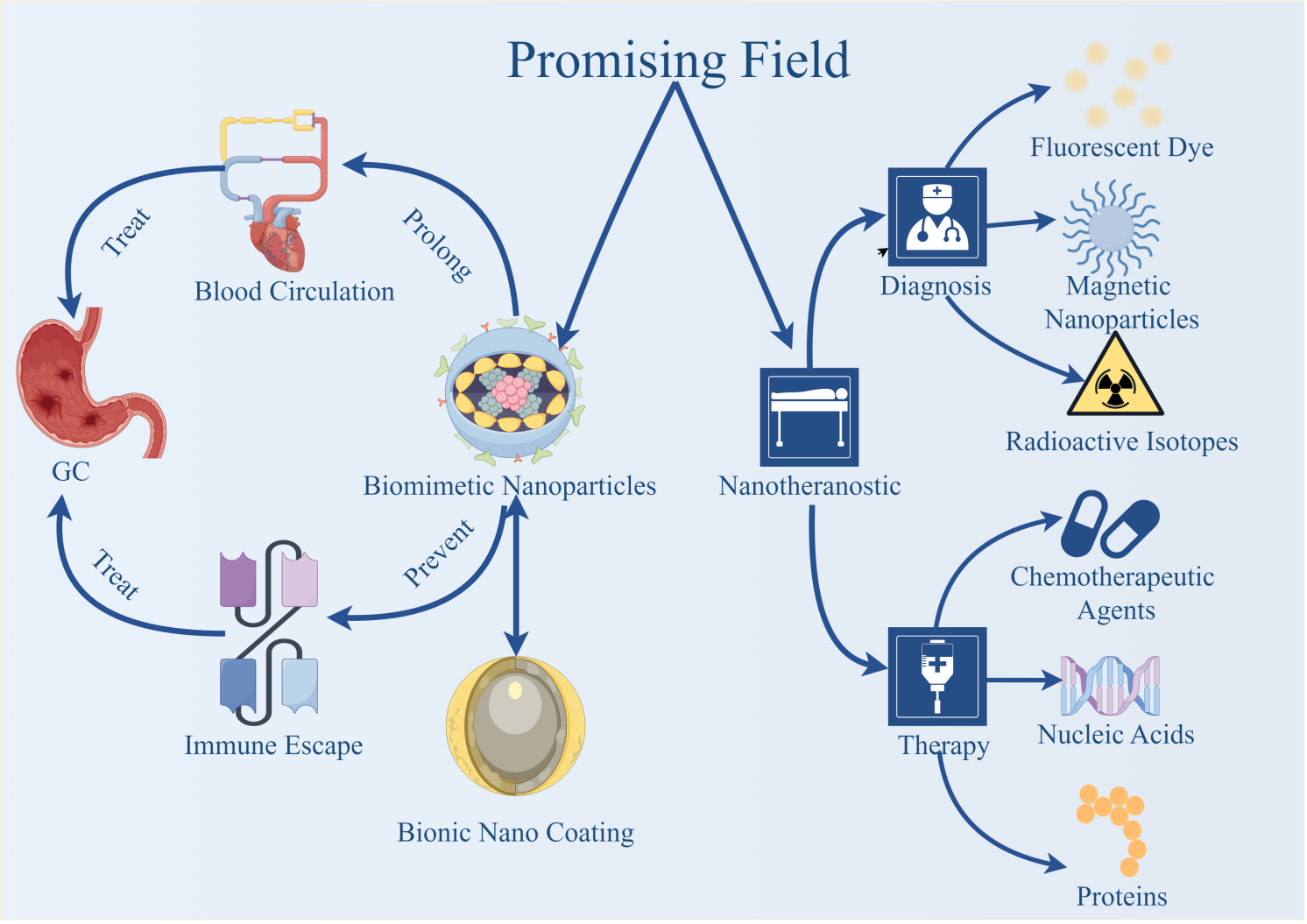


**Figure 4.** Promising Fields in the Development of Nano-drugs for Gastric Cancer
